# Oral Lichen Planus in Children

**DOI:** 10.5005/jp-journals-10005-1043

**Published:** 2009-04-26

**Authors:** Usha Mohan Das, Beena JP

**Affiliations:** 1 Professor and Head, Dean, Department of Pedodontics and Preventive Dentistry, VS Dental College and Hospital, KR Road VV Puram, Bengaluru, Karnataka, India; 2 Lecturer, Department of Pedodontics and Preventive Dentistry, VS Dental College and Hospital, KR Road, VV Puram, Bengaluru Karnataka, India

**Keywords:** Lichen planus, childhood.

## Abstract

Oral lichen planus which is one of the most common oral mucosal diseases in adults, it has been rarely described in children. There are very reports in the literature regarding oral lichen planus in children, here we report a case of intraoral lesions of lichen planus. Lichen planus should be considered in the differential diagnosis of hyperkeratotic or erosive lesions of the oral mucosa in children.

## INTRODUCTION

Lichen planus is a common chronic inflammatory disease of skin and mucous membraness.^1^ Lichen planus is seen most frequently in the middle aged and elderly population 2 and has a female to male ratio of approximately 2:1.^3^ The etiology of lichen planus remains uncertain but many factors have been implicated. Such factors include genetic predisposition, infective agents, systemic diseases, graft-vs.-host disease, drug reactions, and hypersensitivity to dental materials and vitamin deficiencies.^4^ Lichen planus has been associated with several auto-immune diseases, including lupus erythematosus, pemphigus, Sjogren’s syndrome and autoimmune liver disease.^5^,^6^ The pathogenesis of lichen planus is not completely under-stood but a T-lymphocyte infiltrate suggests cell-mediated immunological damage to the epithelium.^7^ Modified Langerhans’ cells and keratinocytes possibly trigger an immune response and the recruitment of T lymphocytes, encouraged by expression of cell-surface adhesion molecules.^4^,^6^ Both CD4 (helper) and CD8 (cytotoxic) cells are present but increasing numbers and activation of the CD8 cells is thought to contribute to the characteristic damage to the basal epithelium.^4^,^7^

Up to six clinical appearances of oral lichen planus have been described,^5^ including reticular, atropic, plaque-like, popular, erosive and bullous types. The characteristic sites involved are the buccal mucosa dorsum of the tongue and less frequently the gingival. There is very little literature on oral lichen planus occurring in childhood.^6^,^8^ This paper reports a case of oral lichen planus in childhood and indicates the importance of considering lichen planus in the differential diagnosis of hyperkeratotic lesions affecting the oral mucosa in childhood.

## CASE REPORT

A 12 years old girl reported to the Department of Pedodontics and Preventive Dentistry at *VS* Dental College and Hospital, Bengaluru, with a chief complaint of burning sensation in her mouth on consuming food for the past 3 months and bilateral pigmentation on the inner part of her cheek.

Medical history and review of system were non contributory. A family history failed to reveal the presence of any similar lesion in the immediate relation.

On examination she appeared to be a healthy 12 years old with no skin rashes. Oral examination showed bilaterally bluish purple striations in the posterior buccal sulci extending onto the buccal mucosa. This was approximately 8 mm × 12 mm in size, flat and nontender on palpation.

**Fig. 1.**
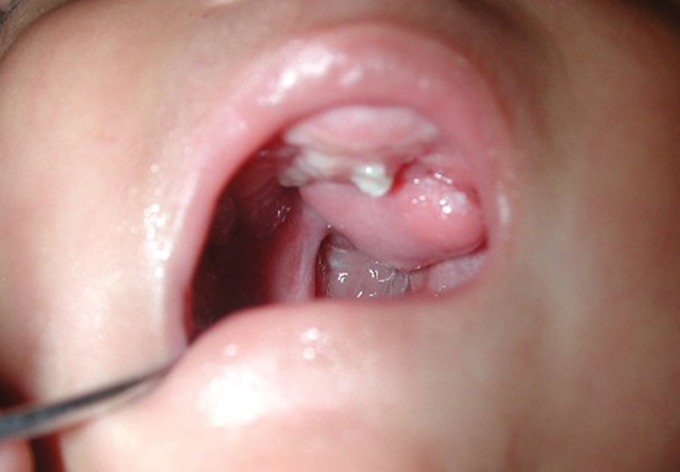
Right buccal mucosa

**Fig. 2.**
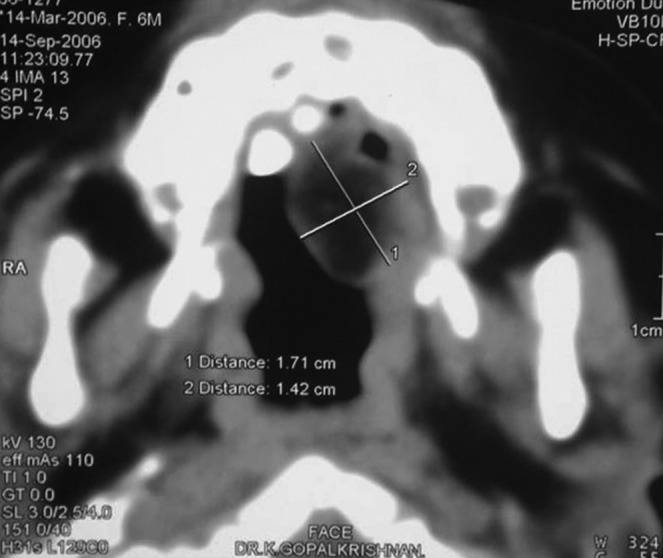
Left buccal mucosa

The dental state was excellent and there were no amalgam restorations. No other mucosal or skin surfaces showed lesional changes.

A provisional diagnosis of reticular lichen planus was made based on clinical examination. Routine haematology, biochemistry and immunology screen were normal except, Haemoglobin which was 9 gm% (normal range 12-14%). Pateint was treated with iron supplements and the haematological tests were repeated, which was followed by an incisional biopsy of the buccal mucosa, histopathological reports confirmed the diagnosis of the lesion to be Lichen Planus.

## TREATMENT DONE

Patient is currently on topical application of 0.05% Tretinoin cream and under weekly review for the first month. Based on the prognosis a decision will be made after a month, whether to start the patient on systemic steroid therapy.

## DISCUSSION

Lichen planus was first described in the literature by Eramus Wilson in 1869,^5^ as predominately a disease of the middle aged or older. There is a limited literature available reporting the occurrences of oral lichen planus in children.^6^,^8^-^17^ Cutaneous lichen planus in childhood is an uncommonly encountered dermatosis^5^,^9^-^14^,^18^,^19^ and is extremely rare in infancy.^5^,^20^ Childhood lichen planus has been documented as a complication of Hepatitis B vaccinations (HBV) where the recombinant proteins of the HBV vaccine, specially the viral S epitope, may trigger a cell-mediated auto-immune response targeted at kertinocytes giving rise to a lichenoid reaction.^21^,^22^ It is also found in association with predisposing conditions such as graft-vs-host disease and chronic active hepatitis C^17^. Studies of children with mucocutaneous lichen planus have shown a very low incidence of oral involvement. Kumar et al, in a series of 25 children with cutaneous lesions, reported only a single patient with oral mucosal lesions^14^ and Kanwar et al, described only 1 patient out of^17^ with mucosal lichen planus involving the lips.^9^

Familial lichen planus has been reported as being uncommon.^8^,^15^,^16^,^18^,^23^ Milligan reported a family history present in 1-2% of cases. Childhood familial lichen planus is said to occur at an early age and with greater severity.^18^

It has been documented that childhood lichen planus is more common in the tropics^14^ and that children of Asian origin may be prone to the condition.^6^,^12^,^18^ Figures from India show a wide range from 1-16 to 11.2%, perhaps supporting the suggestion by Ramsey and Hurley that childhood lichen planus is more common in the tropics.^24^

In summary, although lichen planus in children is rare and oral mucosal involvement, this diagnosis should be considered in children presenting with white lesions of the oral mucosa. Finding from our case report suggest that the condition may present as classical lichen planus without any predisposing medical history nor positive family history.

## CONCLUSION

Although oral lichen planus is considered rare in childhood, the presence of often asymptomatic oral lesions should alert the clinician to such a diagnosis. The case described in this paper highlight the importance of considering lichen planus in the differential diagnosis of hyperkeratotic and erosive lesions of the oral mucosa in childhood.
